# Case Report: Delayed-onset and recurrent infectious endophthalmitis after intravitreal dexamethasone implant

**DOI:** 10.3389/fmed.2026.1962200

**Published:** 2026-10-07

**Authors:** Xinyue Yang, Mengfan Li, Yiwei Zhu, Xiuli Tao, Yan Cheng

**Affiliations:** Department of Ophthalmology, The Second Hospital of Jilin University, Changchun, China

**Keywords:** delayed-onset infection, dexamethasone implant, endophthalmitis, Ozurdex, postoperative recurrence, Staphylococcus epidermidis

## Abstract

**Purpose:**

To report a rare case of delayed-onset infectious endophthalmitis occurring 33 days after an intravitreal dexamethasone implant (Ozurdex®), with recurrence 20 days after complete pars plana vitrectomy and implant removal.

**Methods:**

Case report with clinical findings, diagnostic workup, management, and literature review.

**Results:**

A 51-year-old male with recurrent anterior uveitis received a dexamethasone implant for refractory cystoid macular edema. Thirty-three days post-injection, he developed acute hypopyon and severe vitritis. Emergent 25-gauge pars plana vitrectomy with implant removal was performed. Aqueous humor metagenomic next-generation sequencing (mNGS) confirmed Staphylococcus epidermidis (24,904 reads; 99.96% relative abundance), with no resistance genes detected. Postoperatively, inflammation initially resolved; however, hypopyon recurred 20 days after vitrectomy. A 6-day course of intravenous vancomycin achieved complete resolution. At final follow-up, best-corrected visual acuity improved to 20/33, with no further recurrence.

**Conclusion:**

Endophthalmitis following corticosteroid implants may present with delayed-onset and can recur even after complete surgical removal. Systemic antimicrobial therapy may play a crucial role in eradicating residual infection in such challenging cases.

## Introduction

The intravitreal dexamethasone implant (Ozurdex®) is an established treatment for macular edema (1–3). Endophthalmitis remains a rare but devastating complication, with reported incidences of 0.06% to 0.15% (4, 5). Corticosteroid injections carry a higher risk of endophthalmitis compared to anti-VEGF agents, attributed to the larger 22-gauge needle and local immunosuppression (6–8). The mean time to presentation is typically 2 to 15 days (9–11). However, longer latencies of up to 5 weeks have also been reported (12).

What is notable about this case is the delayed-onset at 33 days post-injection, combined with a postoperative recurrence after complete surgical removal of the implant. This report aims to describe this unusual clinical course and discuss the potential role of systemic antibiotics in management.

## Case description

### Patient information and clinical history

A 51-year-old male with a three-year history of recurrent right anterior uveitis presented to our clinic on August 21, 2025, reporting a 5-day history of right eye swelling and foreign body sensation. His best-corrected visual acuity (BCVA) was 20/50 in the right eye and 20/20 in the left eye. Intraocular pressure (IOP) was 37 mmHg in the right eye and 20 mmHg in the left eye. Slit-lamp examination of the right eye revealed mild mixed conjunctival congestion, keratic precipitates (KP), anterior chamber flare, pharmacologically dilated pupil, mild pigment deposition on the anterior lens capsule, lens opacity, and vitreous opacity. The fundus view was hazy, and the posterior pole retina appeared flat. He was diagnosed with right iridocyclitis and secondary glaucoma and was prescribed topical brinzolamide, brimonidine, carteolol, and atropine.

On August 22, 2025, the patient returned for follow-up. Slit-lamp examination revealed KP, pharmacologically dilated pupil, and mild pigment deposition on the anterior lens capsule. IOP was 9 mmHg in the right eye and 15 mmHg in the left eye. The patient was advised to continue the current topical regimen.

On September 8, 2025, the patient returned for routine follow-up. OCT and FFA were performed to assess disease activity. Given persistent active inflammation, intravenous dexamethasone, topical corticosteroids, and cyclosporine were initiated. His visual acuity initially improved; however, following the death of a family member and a subsequent fracture, he reported discontinuing all medications on November 10, 2025. His condition subsequently deteriorated, and by November 18, 2025, BCVA in the right eye had declined to 20/500.

### Diagnostic assessment and therapeutic intervention

On November 18, 2025, slit-lamp examination revealed mild mixed conjunctival congestion, KP, anterior chamber flare, significant vitreous opacity, and white fibrin deposition on the inferior preretinal area without necrosis. Notably, there was no hypopyon, conjunctival injection, or ocular pain, and the patient had no systemic infectious symptoms (e.g., fever or malaise). As an additional preventive measure, the attending ophthalmologist at our outpatient clinic prescribed topical 0.5% levofloxacin (Cravit®; Santen Pharmaceutical Co., Ltd., Osaka, Japan) four times daily from November 18 to November 20 (for 3 days). OCT confirmed macular edema (Figure 1). Given cystoid macular edema refractory to systemic and topical immunosuppressive therapy, he received an intravitreal dexamethasone implant (0.7 mg; Ozurdex®; Allergan, Irvine, California, USA) in the right eye under sterile conditions on November 20, 2025.

**Figure 1:**
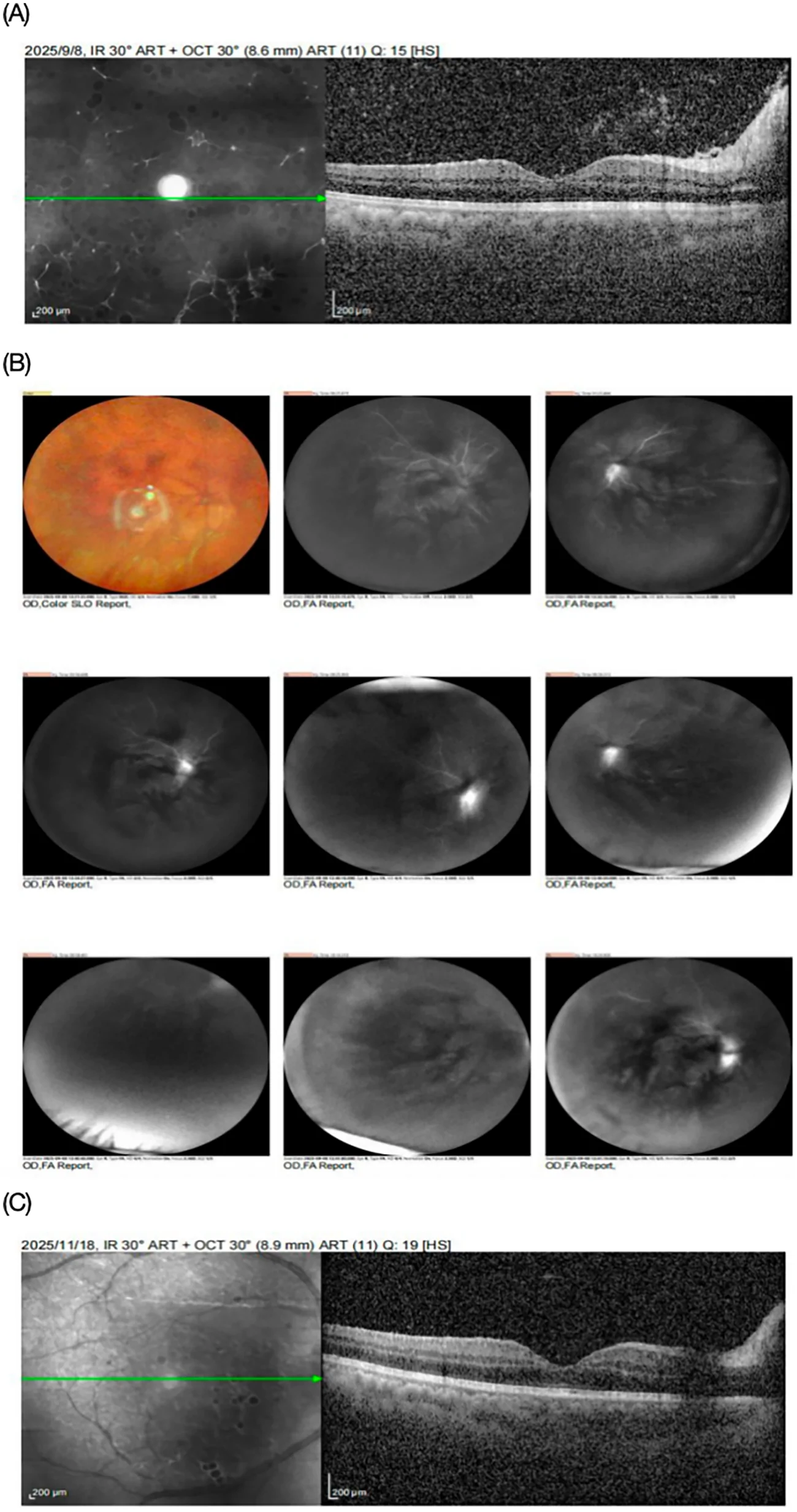
Multimodal imaging of the right eye before dexamethasone implant injection. **(A)** OCT on September 8, 2025, showing persistent cystoid macular edema with intraretinal cystic spaces. **(B)** Fluorescein angiography (FFA) on September 8, 2025 (late phase), revealing diffuse macular leakage and perivascular staining, indicating active inflammation. **(C)** OCT on November 18, 2025, after discontinuation of all medications due to fracture, showing worsening macular edema with increased intraretinal fluid and foveal thickening. These findings demonstrate active inflammatory macular edema refractory to systemic therapy, supporting the subsequent decision for intravitreal dexamethasone implant.

### Follow-up and outcomes

On December 23, 2025 (33 days post-injection), the patient presented with acute, painless vision loss over 3 days. He reported initial visual improvement after the implant but described significant lifestyle disruptions following the bereavement, including poor sleep, excessive alcohol consumption, and dietary changes. On presentation, BCVA was counting fingers at 50 cm, IOP was 10 mmHg, and slit-lamp examination revealed keratic precipitates (++), anterior chamber flare (++), hypopyon, and dense vitreous opacity obscuring the fundus. Color Doppler ultrasonography confirmed dense vitreous opacities without retinal detachment (Figure 2). Laboratory investigations, including HLA-B27 phenotyping, erythrocyte sedimentation rate, plasma D-dimer, and blood glucose, were unremarkable; complete blood count showed mild lymphocytosis (3.6 × 10⁹/L; reference range: 1.1–3.2 × 10⁹/L).

**Figure 2:**
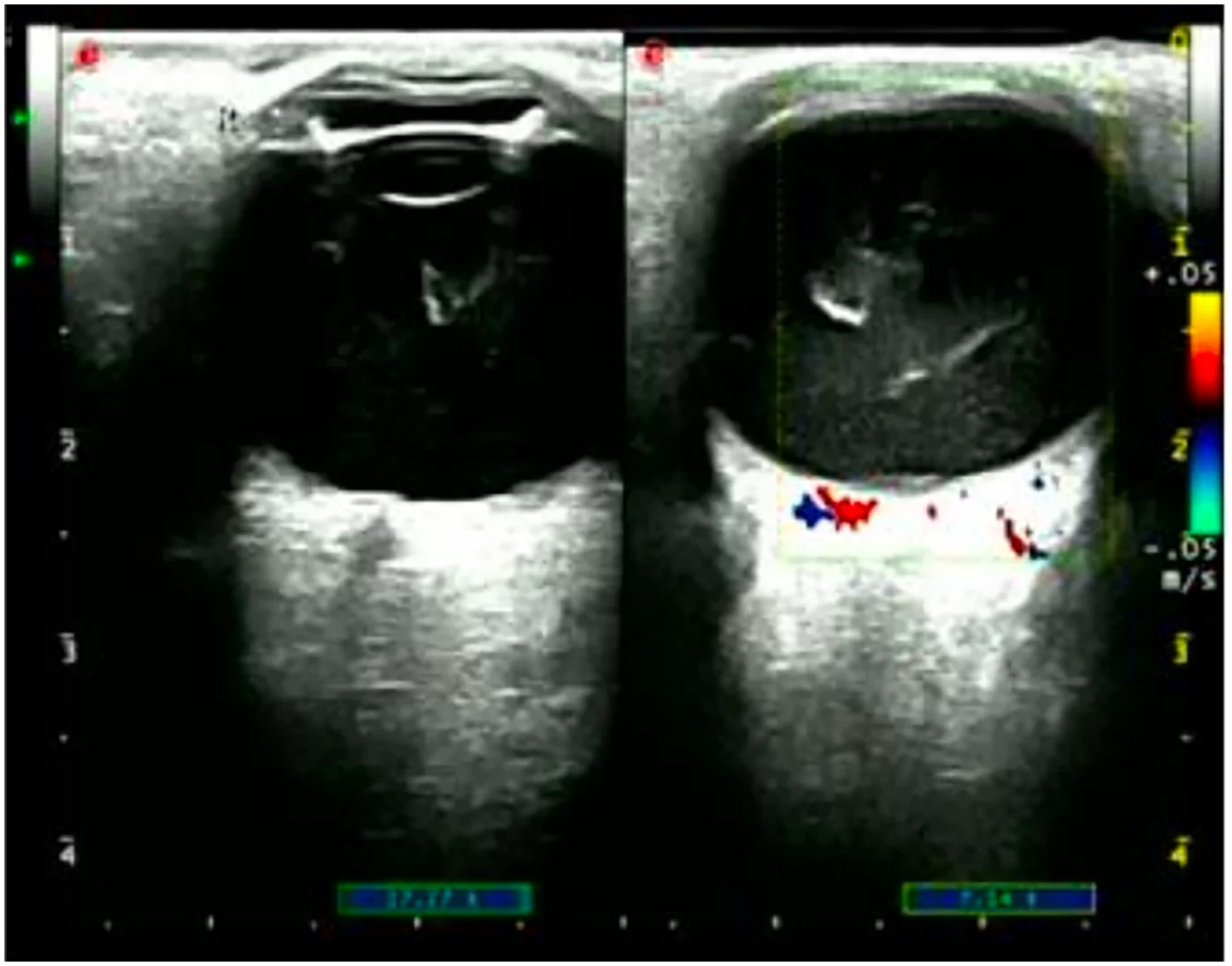
Color Doppler ultrasonography of the right eye at presentation (December 23, 2025; 33 days post-injection). Dense vitreous opacities with membranous and dot-like echoes are visible, along with posterior wall thickening, consistent with severe vitritis and exudative changes. No retinal detachment was observed.

Given the diagnostic uncertainty between a non-infectious uveitic flare and early endophthalmitis, the patient was initially managed in the ophthalmology outpatient clinic with close observation and aggressive topical therapy (TobraDex), without intravitreal antibiotics. However, due to worsening hypopyon and progressive vitritis, an aqueous humor sample was obtained for metagenomic next-generation sequencing (mNGS) on December 25. The mNGS report, available on December 27, confirmed Staphylococcus epidermidis infection, with 24,904 reads (99.96% relative abundance). No resistance genes were detected.Consequently, he was admitted to the Retinal Department on December 27, where preoperative assessments were conducted, and intravenous vancomycin (500 mg every 12 h) was initiated. Emergent 25-gauge pars plana vitrectomy (PPV) was performed on December 28, 2025, during which the dexamethasone implant was completely removed. No intravitreal antibiotics were administered during PPV. This decision was based on two considerations: (1) the clinical presentation was relatively mild compared with the severe, fulminant endophthalmitis commonly encountered in clinical practice, without intense ocular pain, marked corneal edema, or rapid deterioration; and (2) we were concerned about the potential retinal toxicity associated with intravitreal vancomycin injection. A vitreous specimen was obtained intraoperatively. Gram staining of the vitreous specimen revealed numerous leukocytes and Gram-positive cocci. Conventional microbiological testing of the vitreous specimen yielded a smear report on December 28 and a culture/identification report on December 31, both confirming Staphylococcus epidermidis. Intravenous vancomycin was continued for 3 days during hospitalization (December 27–29). At discharge, continuation of the same regimen for an additional 4 days was recommended; however, the patient did not comply with this instruction. On postoperative day 1 (December 29, 2025), BCVA in the right eye improved to 20/50 (uncorrected) and 20/33 (corrected). Fundus photography and fluorescein angiography showed no active inflammation (Figure 3).

**Figure 3:**
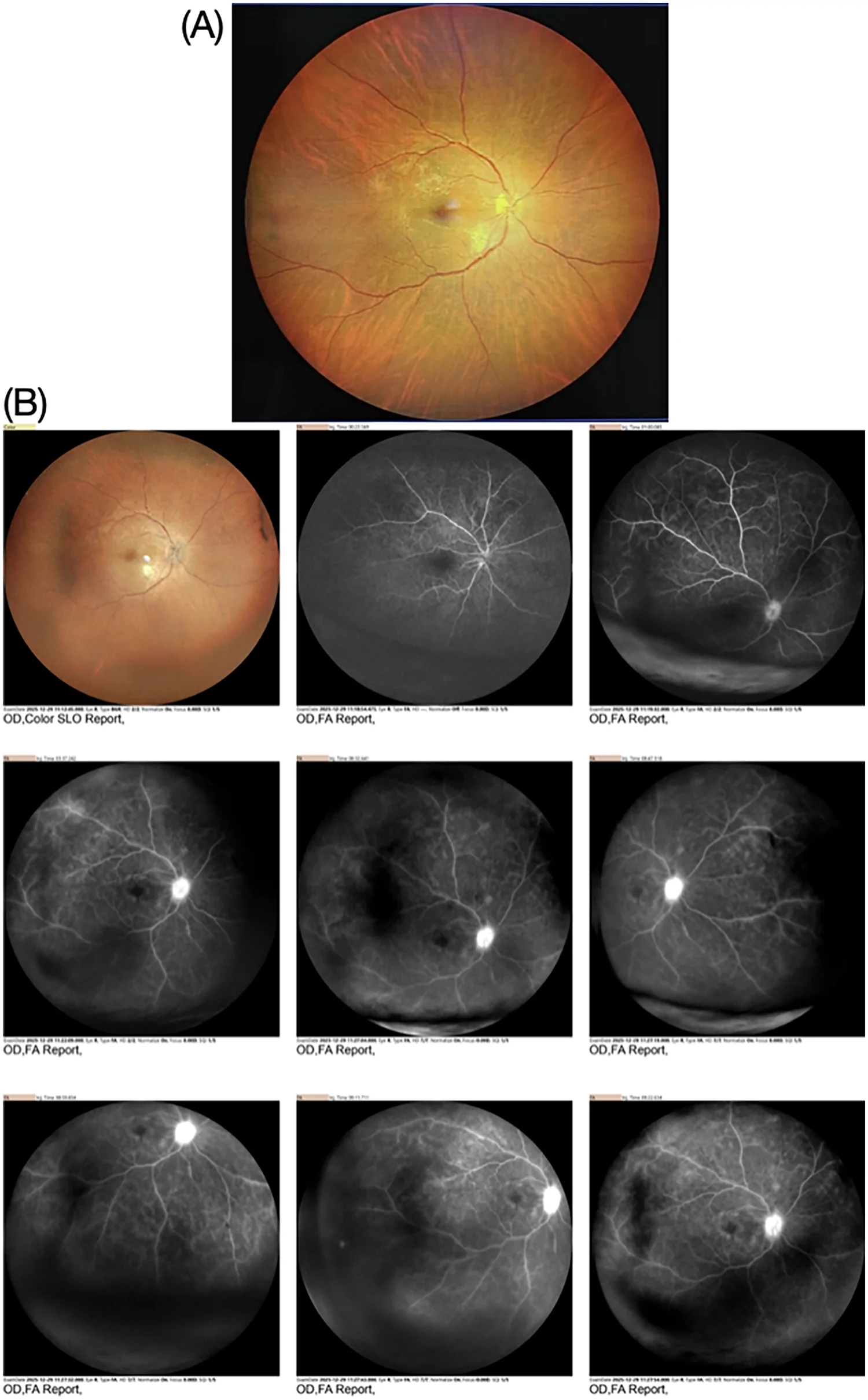
Postoperative fundus photography and fluorescein angiography of the right eye following pars plana vitrectomy. **(A)** Fundus photograph on December 29, 2025 (one day post-vitrectomy), showing a clear vitreous cavity, flat retina, and no obvious hemorrhage or exudation. The optic disc and macula are clearly visible. **(B)** Fluorescein angiography (FFA) on the same day (late phase) showed no evidence of active intraocular inflammation, including absence of macular leakage, vascular wall staining, or optic disc hyperfluorescence. These findings are consistent with successful control of the acute infection.

However, on January 17, 2026 (20 days after vitrectomy), the patient presented with recurrent hypopyon. Slit-lamp examination revealed hypopyon and anterior chamber flare, accompanied by vision decline. Given concern for residual infection, intravenous vancomycin (1 g every 12 h) was initiated. The hypopyon resolved completely within 24 h. On January 18, 2026, examination showed complete hypopyon resolution with residual anterior chamber flare. Intravenous vancomycin was continued for a total of 6 days (January 17–22, 2026). On January 22, 2026, slit-lamp examination revealed no abnormalities, and IOP was 10 mmHg in the right eye and 14 mmHg in the left eye. An anterior chamber tap was performed at that time and yielded negative culture results, which was expected given the resolved state of infection. The patient's last in-person examination was on January 31, 2026, at which BCVA was 20/33, IOP was 12 mmHg in the right eye and 20 mmHg in the left eye, with no other abnormalities and no evidence of recurrence. He subsequently did not return for further care, primarily due to financial constraints and limited health awareness related to his rural residence. A telephone follow-up conducted on September 14, 2026, confirmed the absence of new symptoms or signs of recurrence.

## Discussion

This case presents several unique features: (1) delayed-onset at 33 days post-injection, far exceeding the typical 2- to 15-day window; (2) postoperative recurrence despite complete implant removal via pars plana vitrectomy; and (3) clinical resolution following systemic antibiotic therapy (Figure 4).

**Figure 4:**
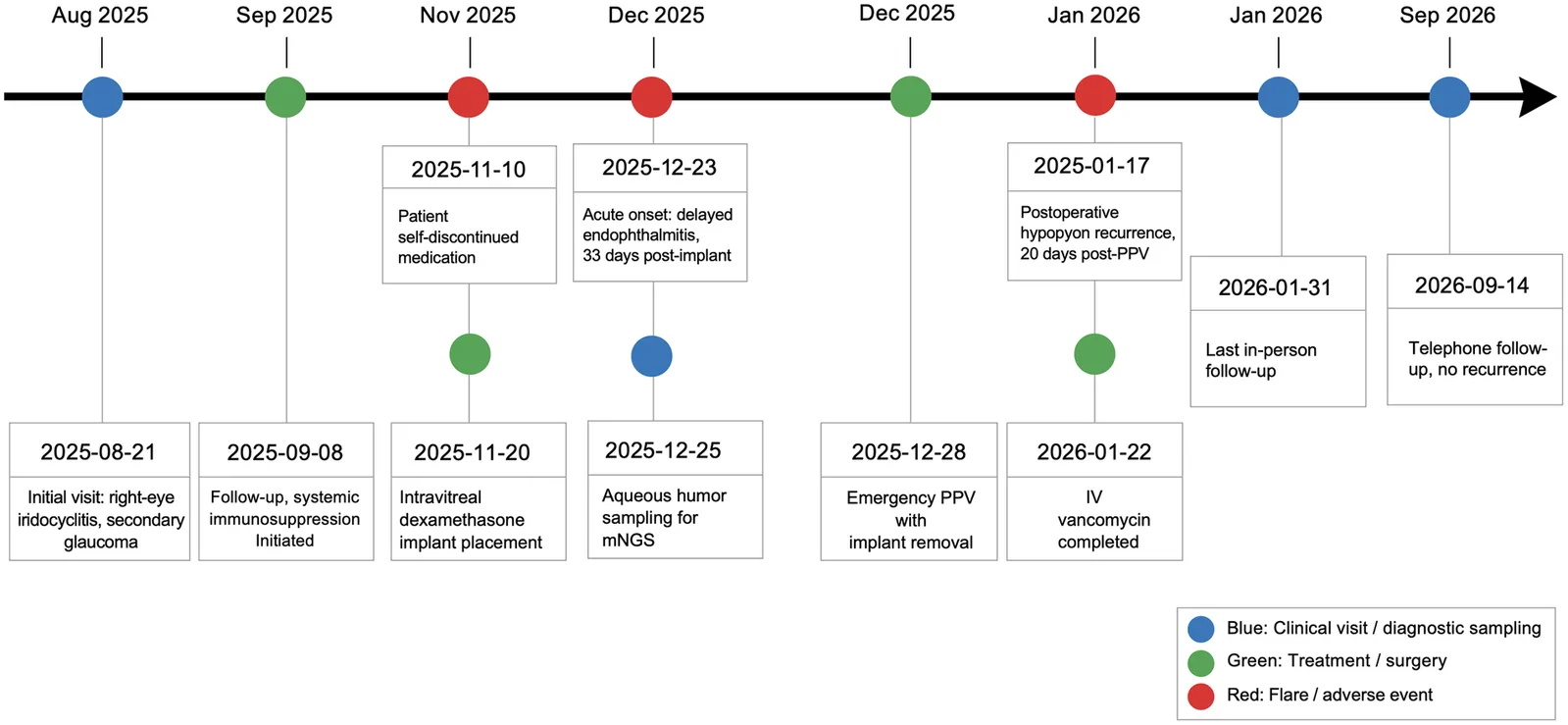
Clinical timeline of key clinical events. This figure chronologically summarizes the patient's clinical course from initial presentation (August 21, 2025) to final telephone follow-up (September 14, 2026). Key milestones include the intravitreal dexamethasone implant placement, onset of delayed-onset endophthalmitis at 33 days post-implant, mNGS confirmation, pars plana vitrectomy (PPV) with implant removal, postoperative recurrence, intravenous vancomycin therapy, and final follow-up assessments. BCVA, best-corrected visual acuity; IOP, intraocular pressure; PPV, pars plana vitrectomy; mNGS, metagenomic next-generation sequencing; IV, intravenous.

The possibility of pre-existing subclinical infection before dexamethasone implantation warrants consideration. However, several lines of evidence argue against this scenario. First, preoperative slit-lamp examination (November 18, 2025) showed active non-infectious uveitis with keratic precipitates, anterior chamber flare, and fibrin deposition—but notably, no hypopyon, a key sign distinguishing infectious from non-infectious inflammation. Second, the patient had no clinical signs of systemic infection (e.g., fever or malaise) prior to injection. Third, had an active infection been present at the time of injection, the potent sustained-release corticosteroid would have suppressed local immunity, likely precipitating explosive, severe endophthalmitis within days rather than weeks. Therefore, the pathogenesis may involve either the exogenous introduction of Staphylococcus epidermidis into the vitreous cavity at the time of injection, or an endogenous mechanism in which impaired systemic and local immunity permitted the slow proliferation of pre-existing commensal organisms. The locally immunosuppressive environment created by the dexamethasone implant, compounded by the patient's abrupt withdrawal of systemic immunosuppressants on November 10, 2025, likely played a critical permissive role in this process, ultimately manifesting as acute endophthalmitis at 33 days post-injection.

The prolonged incubation period may be attributed to the local immunosuppressive effect of the sustained-release dexamethasone implant, which dampens inflammation and allows slow bacterial proliferation. Staphylococcus epidermidis is the most common pathogen in such cases, accounting for approximately 40% of culture-positive cases (13, 14).

The postoperative recurrence observed in this case challenges the assumption that implant removal alone suffices to eradicate infection (9, 15, 16). At the time of recurrence (January 17, 2026), the differential diagnosis included: (1) infectious endophthalmitis recurrence; (2) recurrent non-infectious uveitis; and (3) sterile inflammation (6). Several clinical features favored an infectious etiology. First, the recurrence presented with a new hypopyon and anterior chamber flare, which are atypical for a simple uveitic flare in a patient previously managed with immunosuppressants. Second, the patient initially presented to a local hospital on January 17, 2026, and was unable to travel to our institution. Following remote consultation between the local physicians and our team, empirical intravenous vancomycin was initiated, and the hypopyon resolved completely within 24 h. When the patient subsequently presented to our hospital on January 22, examination revealed no abnormalities in either eye, and an anterior chamber tap performed at that time yielded negative culture results, which may be partly attributable to the prior administration of systemic vancomycin. The rapid resolution of the hypopyon following systemic vancomycin, without the addition of aggressive topical or systemic corticosteroids, is more consistent with an infectious etiology than with sterile inflammation or a non-infectious uveitic flare, which would typically require corticosteroid escalation. However, immune dysregulation may also have contributed to the recurrence. The patient had a history of recurrent uveitis and had abruptly discontinued systemic immunosuppressants, which may have impaired local immune surveillance, thereby permitting the proliferation of residual organisms. Nevertheless, given the prompt response to vancomycin, residual infection remains the most plausible explanation, with immune dysregulation as a potential contributing factor. A plausible mechanism for persistent residual infection is biofilm formation, as it is a well-established virulence mechanism of S. epidermidis in device-associated infections and has been implicated in recurrent endophthalmitis (17); metabolically quiescent persister cells within biofilms can also survive high antibiotic concentrations and repopulate once therapy is reduced (18). Although systemic vancomycin penetrates the vitreous less readily than agents such as moxifloxacin, it can reach concentrations exceeding the minimum inhibitory concentration for common gram-positive pathogens under conditions of blood-retinal barrier disruption (19). Collectively, these data support the use of systemic vancomycin as an adjunctive therapy when local antibiotic delivery is insufficient to eradicate residual biofilm-associated infection. Therefore, although microbiological confirmation was not obtained at the exact moment of recurrence, the clinical course and response to targeted antimicrobial therapy strongly suggest a residual infectious etiology.

### Comparison with similar and contrasting cases

Unlike most reported cases of dexamethasone implant-associated endophthalmitis, which typically present within 2–15 days (9–11), our case occurred at 33 days. Although Enger et al. reported delayed onset up to 5 weeks (12), the recurrence after complete implant removal is, to our knowledge, rarely documented. This distinguishes our case from those managed without implant removal (9) or those caused by Brevibacterium casei (15).

### Strengths and limitations

The main strength of this case is the detailed documentation of both the delayed-onset and the postoperative recurrence, supported by microbiological confirmation via culture and mNGS. As a single case report, the generalizability of these findings is limited. Additionally, because no anterior chamber tap was performed at the time of recurrence (the patient was at a local hospital), definitive confirmation of the same pathogen is precluded; however, the prompt resolution with systemic vancomycin strongly suggests a residual infectious etiology. Furthermore, the optimal duration of systemic antibiotic therapy remains uncertain based on a single case, and the follow-up duration was relatively short due to socioeconomic factors, although a telephone follow-up at 8 months confirmed no recurrence.

Perioperative antimicrobial management merits further discussion. In this case, no intravitreal antibiotics were administered during PPV. This decision was guided by the relatively mild clinical presentation and concerns regarding the retinal toxicity of intravitreal vancomycin. Experimental studies have demonstrated that intravitreal vancomycin at doses up to 2 mg is generally non-toxic in phakic and aphakic-vitrectomized eyes; however, toxic retinopathy has been reported with intravitreal injection at usual concentrations, and the safe clinical single-shot dose has been suggested to be less than 1 mg (20, 21). Consequently, we elected to rely on systemic vancomycin alone. The rationale for intravenous vancomycin is supported by pharmacokinetic evidence: although systemic penetration into the vitreous is generally limited, vitreous concentrations in aphakic and aphakic-vitrectomized eyes after intravenous administration can exceed the minimum inhibitory concentrations for common gram-positive pathogens that cause endophthalmitis (22, 23). In the present case, the patient had undergone PPV (resulting in an aphakic-vitrectomized state), and the infection was caused by Staphylococcus epidermidis, a gram-positive organism highly susceptible to vancomycin. The patient received intravenous vancomycin (500 mg every 12 h) for 3 days during hospitalization (December 27–29). At discharge, continuation for an additional 4 days was recommended, but the patient did not comply. No adverse events related to intravenous vancomycin were observed during hospitalization, and renal function was monitored and remained normal.

### Take-away lessons

This case raises several important clinical questions. First, should systemic antibiotics be routinely considered as an adjunct to vitrectomy in implant-associated endophthalmitis, even after complete implant removal? Our patient developed recurrent hypopyon 20 days after vitrectomy despite complete implant removal, and the recurrence resolved promptly with intravenous vancomycin. Although microbiological confirmation of the recurrent infection was not available, this clinical response strongly suggests that surgical eradication alone may be insufficient, possibly due to residual bacteria sequestered in the vitreous base or adherent to adjacent structures (24, 25).

Second, what is the optimal duration of systemic therapy when postoperative recurrence occurs? Our patient received a 6-day course of intravenous vancomycin, which achieved complete resolution through the last follow-up. However, whether this suffices or a longer course is needed remains uncertain.

Third, this case highlights the clinical utility of mNGS in guiding timely and targeted therapy. In this case, mNGS of the aqueous humor provided a definitive result on December 27, whereas conventional smear and culture of the intraoperative vitreous sample were not available until December 28 and December 31, respectively. Although conventional testing ultimately confirmed the same pathogen, the rapid turnaround of mNGS enabled the early initiation of targeted intravenous vancomycin on December 27, avoiding prolonged empirical broad-spectrum antibiotic therapy. Given the characteristically low positivity rates of conventional smear and culture for endophthalmitis, mNGS provides a critical diagnostic advantage. Without mNGS, management would have relied on empirical treatment for at least 4 additional days, potentially delaying targeted therapy and increasing the risk of inappropriate antibiotic use.

We conclude that dexamethasone implant-associated endophthalmitis may present with delayed-onset and can recur even after complete surgical removal. Clinicians should maintain a high index of suspicion for atypical infections in patients receiving corticosteroid implants, and systemic antimicrobial therapy may play a crucial role in eradicating residual infection.

## Patient perspective

The patient was informed about the nature of his condition and the planned interventions. He provided written informed consent for the publication of this case report and accompanying images. He expressed gratitude for the care received and hoped that sharing his experience could help other patients with similar conditions.

## Data Availability

The datasets presented in this study can be found in online repositories. The names of the repository/repositories and accession number(s) can be found in the article/Supplementary Material.
